# Variants of cancer susceptibility genes in Korean *BRCA1/2* mutation-negative patients with high risk for hereditary breast cancer

**DOI:** 10.1186/s12885-017-3940-y

**Published:** 2018-01-16

**Authors:** Ji Soo Park, Seung-Tae Lee, Eun Ji Nam, Jung Woo Han, Jung-Yun Lee, Jieun Kim, Tae Il Kim, Hyung Seok Park

**Affiliations:** 1 Hereditary Cancer Clinic, Cancer Prevention Center, Yonsei Cancer Center, Yonsei University College of Medicine, Seoul, Republic of Korea; 2 Department of Laboratory Medicine, Yonsei University College of Medicine, Seoul, Republic of Korea; 3 Department of Obstetrics and Gynecology, Institute of Women’s Life Medical Science, Women’s Cancer Clinic, Yonsei University College of Medicine, Seoul, Republic of Korea; 4 Department of Pediatrics, Yonsei University College of Medicine, Seoul, Republic of Korea; 5 Department of Laboratory Medicine, Soonchunhyang University School of Medicine, Seoul, Republic of Korea; 6 Division of Gastroenterology, Department of Internal Medicine, Yonsei University College of Medicine, Seoul, Republic of Korea; 7 Department of Surgery, Yonsei University College of Medicine, 50-1 Yonsei-ro, Seodaemun-gu, Seoul, 03722 Republic of Korea

**Keywords:** Breast neoplasms, Neoplastic Syndromes, Hereditary, Beyond BRCA1/2, Multigene panel, Next generation sequencing

## Abstract

**Background:**

We evaluated the incidence and spectrum of pathogenic and likely pathogenic variants of cancer susceptibility genes in *BRCA1/2* mutation-negative Korean patients with a high risk for hereditary breast cancer using a comprehensive multigene panel that included 35 cancer susceptibility genes.

**Methods:**

Samples from 120 patients who were negative for *BRCA1/2* mutations, but had been diagnosed with breast cancer that was likely hereditary, were prospectively evaluated for the prevalence of high-penetrance and moderate-penetrance germline mutations.

**Results:**

Nine patients (7.5%) had at least one pathogenic or likely pathogenic variant. Ten variants were identified in these patients: *TP53* in two patients, *PALB2* in three patients, *BARD1* in two patients, *BRIP1* in two patients, and *MRE11A* in one patient. We also identified 30 types of 139 variants of unknown significance (VUS). High-penetrance germline mutations, including *TP53* and *PALB2,* tended to occur with high frequency in young (< 35 years) breast cancer patients (4/19, 21.1%) than in those diagnosed with breast cancer at ≥35 years of age (1/101, 1.0%; *p* = 0.003).

**Conclusions:**

These combined results demonstrate that multigene panels offer an alternative strategy for identifying veiled pathogenic and likely pathogenic mutations in breast cancer susceptibility genes.

**Electronic supplementary material:**

The online version of this article (10.1186/s12885-017-3940-y) contains supplementary material, which is available to authorized users.

## Background

The identification of *BRCA1* and *BRCA2* germline mutations as predictors of cancer susceptibility significantly improved the diagnosis and prevention of hereditary breast and ovarian cancers (HBOC). Recent advances in genetic testing have enabled the discovery of novel genes that increase the risk of cancer in patients with familial predisposition. Multiple research laboratories have evaluated these cancer-associated mutations in patients who are negative for *BRCA1/2* mutations, but still have a high risk of HBOC. These efforts have identified mutations in moderate-risk genes, such as *ATM, BRIP1, CHEK2, BARD1, MRE11A, NBN, RAD50, RAD51*, and *XRCC2*, as well as those in high-penetrance genes, including *TP53, PTEN, STK11, CDH1,* and *PALB2*, have been reported across diverse ethnic populations [1].

Next generation sequencing (NGS) can provide detailed genetic information via multi-gene panel assays [2]. However, the application of NGS multigene panel test in a clinical setting represents a considerable challenge. It is necessary to not only validate this novel technique, but also to select candidate susceptibility genes. Furthermore, mutations indicative of cancer susceptibility vary across ethnicities; therefore, it is important to understand the clinical and genetic characteristics of multiple susceptibility genes identified by NGS multigene panels in each ethnic population.

In this study, we used comprehensive multigene panels that included 35 known or suspected cancer susceptibility genes to examine *BRCA1/2* mutation-negative Korean patients who had clinical features indicative of hereditary breast cancer. We also investigated the feasibility of multigene panel testing for Korean patients, and evaluated potential clinicopathological risk factors related to germline mutations other than *BRCA1/2*.

## Methods

### Study population

The study population included 182 Korean *BRCA1/2* mutation-negative breast cancer patients with a familial predisposition who were referred to the Cancer Prevention Center, Yonsei Cancer Center, Seoul, Korea between March 1, 2015 and November 11, 2016. Sixty-two patients opted to not participate. Finally, a total of 120 patients were enrolled in the study. Suspected clinical features of hereditary breast cancer were defined as follows: (1) at least one case of breast or ovarian cancer in first- or second-degree relatives; (2) a first diagnosis of breast cancer before age 40; (3) bilateral breast cancer; and (4) co-diagnosis of breast and ovarian cancers in the same patient.

### Panel-based mutation analysis

Germline DNA was extracted from the participants’ peripheral blood samples. We used a customized targeted capture sequencing panel (OncoRisk®, Celemics, Seoul, Korea) which included all coding sequences and intron-exon boundaries of the coding exon from 35 cancer predisposition genes (*BRCA1, BRCA2, PALB2, BARD1, BRIP1, RAD51C, RAD51D, RAD50, NBN, MRE11A, ATM, CHEK2, TP53, PTEN, APC, BLM, BMPR1A, CDH1, CDK4, CDKN2A, EPCAM, MEN1, MLH1, MSH2, MSH6, MUTYH, PMS2, POLE, PRSS1, RET, SLX4, SMAD4, STK11, VLH*, and *WT1*). Products with each capture reaction were sequenced by 100 base pair paired-end reads on a MiSeq platform (Illumina, San Diego, CA). High-quality sequencing data with an average depth of 500−1000 folds were obtained.

We identified all single base pair substitutions, insertion-deletions, and copy number variants (CNVs) in each gene. Split-read-based detection of large insertions and deletions was conducted using the Pindel and Manta algorithms. CNVs detected by ExomeDepth software [3] were further crosschecked with our custom pipelines, which retrieved base-level depth of coverage for each binary alignment map (BAM) file using SAMtools software (http://samtools.sourceforge.net) and normalized the depths in the same batch (Additional file 1: Figure S1). All likely deleterious mutations were validated by Sanger sequencing, and all possible large rearrangements were confirmed by the multiplex ligation-dependent probe amplification (MLPA) method (Additional file 1: Figure S2).

Genetic variants were classified using a five-tier system following guidelines from the American College of Medical Genetics and Genomics (ACMG) as follows: pathogenic, likely pathogenic, variants of unknown significance (VUS), likely benign, or benign/polymorphism [4]. We used the Sorting Intolerant From Tolerant (SIFT, http://sift.bii.a-star.edu.sg/) and Polymorphism Phenotyping-2 (PolyPhen-2, http://genetics.bwh.harvard.edu/pph2) to generate in silico predictions of several of the identified nonsynonymous variants. Using large rearrangements of exons, pathogenic and likely pathogenic variants were considered as mutations, for consistency with previous studies [5].

## Results

**Fig. 1:**
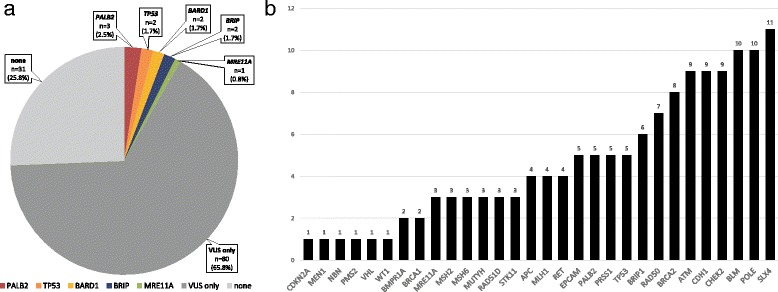
**a** Percentage of patients with pathogenic or likely pathogenic mutations corresponding with each gene. **b** Number of patients with variants of uncertain significance (VUS) for each gene (*n* = 120 patients total)

Baseline characteristics of the patients are presented in Additional file 2: Table S1. A total of 7.5% (9/120) of patients were found to carry at least one pathogenic or likely pathogenic variant. A total of ten gene variants (Fig. 1a) were identified in nine patients: *TP53* in two patients, *PALB2* in three patients, *BARD1* in two patients, *BRIP1* in two patients, and *MRE11A* in one patient. We detected a large deletion from exon 2−9 in the *TP53* gene, and the other pathogenic variants identified were as follows: *PALB2* (c.3267_3268delGT, p.Phe1090SerfsTer6, rs587781890; c.2257C > T, p.Arg753Ter, rs180177110; and c.695delC, p.Gly232ValfsTer6); *BARD1* (c.1345C > T, p.Gln449Ter); *BRIP1* (c.1066C > T, p.Arg356Ter, rs730881633; and exon 5–6 deletion); and *MRE11A* (c.1773_1774delAA, p.Gly593LysfsTer4). Likely pathogenic variants were found in *TP53* (c.733G > A, p.Gly245Ser, rs28934575). Pathogenic variants in *PALB2* and *MRE11A* were identified in a 34-year-old patient who was co-diagnosed with breast and gastric cancer (Table 1). Three of the pathogenic variants identified in this study were not reported previously.

**Table 1:** Characteristics of patients with pathogenic or likely pathogenic variants

Case number	Site/histology of breast cancer	Breast cancer subtype	Breast cancer stage (AJCC 7th ed)	Concomitant cancers	Affected gene	Nucleotide change	Amino acid change	dbSNP	Variant effect	Family cancer history (family member, age)	MAF by ExAC (*n* = 60,704)	MAF by ExAC Asian (*n* = 12,583)	MAF by KRGDB(*n* = 622)	Confirmation method	Pathogenicity	Reference
1	L/IDC	ER+/PR+/HER2-	IIA	–	*TP53*	exon2–9 deletion	N/A	–	Large deletion	Breast ca (mother, 32)	N/A	N/A	N/A	MLPA	Pathogenic	
2	B/IDC	ER+/PR+/HER2-	IIA	–	*PALB2*	c.3267_3268delGT	p.Phe1090SerfsTer6	rs587781890	Frameshift	Breast ca (aunt, 47), Colon ca (GF, 60), Stomach ca (GM, 60)	–^**^	–^**^	–^**^	Sanger sequencing	Likely pathogenic	
3	R/IDC	ER+/PR+/HER2-	IIB	AoV	*PALB2*	c.2257C > T	p.Arg753Ter	rs180177110	Nonsense	Breast ca (sister, 53)	3.29 × 10^−5^	–^**^	–^**^	Sanger sequencing	Pathogenic	
4*	L/poorly differentiated	TNBC	IA	Stomach	*PALB2*	c.695delG	p.Gly232ValfsTer6	–	Frameshift	Stomach ca (GF, 90), Liver ca (uncle, 60)	–^**^	–^**^	–^**^	Sanger sequencing	Likely pathogenic	
4*	L/poorly differentiated	TNBC	IA	Stomach	*MRE11A*	c.1773_1774delAA	p.Gly593LysfsTer4	–	Frameshift	Stomach ca (GF, 90), Liver ca (uncle, 60)	–^**^	–^**^	–^**^	Sanger sequencing	Likely pathogenic	
5†	L/mucinous	TNBC	IA	–	*BARD1*	c.1345C > T	p.Gln449Ter	–	Nonsense	Breast ca (sister1, 67; sister2, 47)	–^**^	–^**^	–^**^	Sanger sequencing	Likely pathogenic	
6†	L/IDC	ER+/PR-/HER2-	IIA	–	*BARD1*	c.1345C > T	p.Gln449Ter	–	Nonsense	Breast ca (sister1, 67; sister2, 58)	–^**^	–^**^	–^**^	Sanger sequencing	Likely pathogenic	
7	L/IDC	ER-/PR-/HER2+	IA	–	*BRIP1*	exon5–6 deletion	N/A	–	Largedeletion	Ovarian ca (mother, 35)	N/A	N/A	N/A	MLPA	Pathogenic	
8	R/IDC	ER-/PR-/HER2+	IA	Cervix uteri	*BRIP1*	c.1066C > T	p.Arg356Ter	rs730881633	Nonsense	Breast ca (sister, 40)	–^**^	–^**^	–^**^	Sanger sequencing	Likely pathogenic	
9	B/IDC	ER-/PR-/HER2+	IIA	–	*TP53*	c.733G > A	p.Gly245Ser	rs28934575	Missense	Stomach ca (father, 56); Pancreatic ca (father, 73)	8.24 × 10^−6^	–^**^	–^**^	Sanger sequencing	Likely pathogenic (Table S2)	[23]

Abbreviation: AJCC, American Joint Committee on Cancer; AoV, ampulla of Vater; B: bilateral; ca: cancer; dbSNP, single nucleotide polymorphism database; DCIS, ductal carcinoma in situ; ER, estrogen receptor; ExAC, Exome Aggregation Consortium; HER2, human epidermal growth factor receptor 2; IDC, invasive ductal carcinoma; KRGDB, Korean Reference Genome database; L, left; N/A, not assessable; MAF, minor allele frequency; MLPA, multiplex ligation-dependent probe amplification; Polyphen, Polymorphism Phenotyping-2; PR, progesterone receptor; R, right; SIFT, Sorting Intolerant From Tolerant; TNBC, triple negative breast cancer

*Case 4 had pathogenic variants in *PALB2* and *MRE11A*. ^†^Case 5 and Case 6 are siblings. **There was no case with the relevant variant in the databases with respect to the general population

A total of 87 patients (72.5%) had at least one VUS (median, 1; range, 0–3). A total of 139 VUS were identified in 30 cancer susceptibility genes, including *SLX4* (*n* = 11), *BLM* (*n* = 10), *POLE* (n = 10), *ATM* (*n* = 9), *CDH1* (n = 9), *CHEK2* (n = 9), *BRCA2* (*n* = 8), *RAD50* (*n* = 7), *BRIP1* (*n* = 6), *EPCAM* (*n* = 5), *PALB2* (n = 5), *PRSS1* (n = 5), *TP53* (n = 5), *APC* (*n* = 4), *MLH1* (n = 4), *RET* (n = 4), *MRE11A* (*n* = 3), *MSH2* (n = 3), *MSH6* (n = 3), *MUTYH* (n = 3), *RAD51D* (n = 3), *STK11* (n = 3), *BMPR1A* (*n* = 2), *BRCA1* (n = 2), *CDKN2A* (n = 1), *MEN1* (n = 1), *NBN* (n = 1), *PMS2* (n = 1), *VHL* (n = 1), and *WT1* (n = 1) (Fig. 1b).

First diagnosis of breast cancer at a relatively young age (<35 years) was correlated with pathogenic or likely-pathogenic variants in high-penetrance cancer susceptibility genes. Pathogenic variants in high-penetrance genes were detected in 21.1% (4/19) of these patients, which was significantly higher than that for patients who were first diagnosed with breast cancer at age ≥ 35 years (1/101, 1.0%, *p* = 0.003) (Table 2).

**Table 2:** Association between the clinicopathological features of suspected hereditary breast cancer and the pathogenic or likely pathogenic variants of non-*BRCA* cancer predisposition genes (*n* = 120 patients)

Clinicopathological features		High-penetrance mutations		Moderate-penetrance mutations		None or VUS		
		Number ofpatients	%	Number ofpatients	%	Number ofpatients	%	*p*-value
Breast cancer site								
	Bilateral	2	18.2	0	0	9	81.8	0.106*
	Unilateral	3	2.8	4	3.7	102	93.5	
Breast cancer subtype (*n* = 117, excluding patients with unknown breast cancer subtypes)								
	TNBC	0	0	1	4.5	21	95.5	>0.99*
	hormone + and/or HER2+	4	4.2	3	3.2	88	92.6	
Concomitant diagnosis with ovarian cancer								
	Yes	0	0	0	0	3	100	>0.99*
	No	5	4.3	4	3.4	108	92.3	
Age at first diagnosis of breast cancer								
	< 35 years	4	21.1	0	0	15	78.9	0.003*
	≥ 35 years	1	1.0	4	4.0	96	95.0	
Family history of young (< 50 years old at diagnosis) breast and/or ovarian cancer patients within 2nd degree family								
	Yes	2	6.3	3	9.4	27	84.3	0.053*
	No	3	3.4	1	1.1	84	95.5	

Abbreviations: HER2, human epidermal growth factor receptor 2; TNBC, triple negative breast cancer; VUS, variant of unknown significance. *Analyzed using Fisher’s exact test

## Discussion

Previous studies using multigene panel tests identified cancer susceptibility genes in 2.1−16.8% of *BRCA1/2* mutation-negative patients [5–11]. Our tests of high-penetrance genes identified a large exon deletion in *TP53*, and pathogenic and likely pathogenic variants in *TP53* and *PALB2* (Table 1). We also identified a frameshift mutation of *MRE11A* c.1773_1774delAA (p.Gly593LysfsTer4) in a patient with a *PALB2* mutation. The MRE11 protein functions in non-homologous end-joining and homologous recombination, which occur during the repair of double-stranded DNA breaks [12]. Therefore, the risk for patients with concurrent dysfunction in *PALB2* and *MRE11A* is unclear and should be assessed in future studies*.* Because the two frameshift variants in *PALB2* (c.3267_3268delGT, p.Phe1090SerfsTer6, rs587781890; and c.695delG, p.Gly232ValfsTer6) were not found in the control group, the variants met the criteria to be likely pathogenic according to the ACMG guideline (PVS1 and PM2) (Table 1) [4]. One nonsense variant in *PALB2* (c.2257C > T p.Arg753Ter, rs180177110) had a higher prevalence in affected patients compared to the control group [odds ratio (OR), 127.0; 95% confidence interval (CI), 14.1–1140.1; *p* < 0.0001]. Therefore, this variant conformed to the criteria to be classified as pathogenic according to ACMG guidelines (PVS1 and PS4) (Table 1) [4]. In addition, a missense variant in *TP53*, c.733G > A (p.Gly245Ser, rs28934575) was classified as a pathogenic or likely pathogenic variant in the ClinVar database (http://www.ncbi.nlm.nih.gov/clinvar/), and met the criteria for a likely pathogenic variant according to the ACMG guidelines (PM2, PM5, PP2, PP3, and PP5) (Additional file 2: Table S2) [4].

Pathogenic or likely pathogenic variants also were detected in *BRCA1*-associated RING domain 1 (*BARD1*) and *BRCA1*-interacting protein C-terminal helicase 1 (*BRIP1*). *BARD1* and *BRIP1* encode proteins that interact with the BRCA1 protein during the repair of DNA double- stranded break, and pathogenic variants of these genes have been investigated [13]. However, there is a controversy as to whether these rare variants are clinically associated with a risk of breast cancer [11, 14]. In a previous study that screened for *BRIP1* mutations among 235 Korean patients with *BRCA1/2* mutation-negative high-risk breast cancers using fluorescent-conformation sensitive gel electrophoresis (F-CSGE), there was no case of a protein-truncating *BRIP1* mutation, which suggests that the prevalence of *BRIP1* mutations is likely to be low in the Korean population [15].

Cell cycle checkpoint kinase 2 (*CHEK2*) is a well-established moderate-penetrance breast cancer gene. Several studies have shown that essentially no case of *CHEK2* (c.1100delC) was observed in Asian populations, in contrast to the observed prevalence in European populations [16–19]. Liu and colleagues reported that the *CHEK2* c.1111C > T (p.His371Tyr, rs531398630) variant was observed in 4.24% (5/118) of Chinese familial breast cancer cases without *BRCA1/2* mutations, and was associated with dysfunctional phosphorylation of T68 in the SQ/TQ rich domain, which is an activation point following DNA damage [18]. We also identified *CHEK2* c.1111C > T variants in 2.5% (3/120) of Korean breast cancer patients without *BRCA1/2* mutations (Additional file 2: Table S2). Population-based investigations are required to establish the prevalence of this variant, especially in Asian patients. We identified the *CHEK2* c.908 + 2delT variant in one patient, and it was classified as likely pathogenic according to the ACMG guideline (Additional file 2: Table S2). However, we did not classify this variant as a positive result because the experimental study was not sufficient.

In the current study, clinically important likely pathogenic or pathogenic variants of high-penetrance genes were identified in only five (4.2%) patients (*TP53* in two patients*,* and *PALB2* in three patients). These variants were identified in 4 of 19 patients (21.1%) with early-onset breast cancer (< 35 years old at onset) (Table 2). A previous study identified cancer susceptibility mutations in 11% of *BRCA1/2*-negative patients with early-onset breast cancer (diagnosed at <40 years of age) [20]. Considering the frequency of pathogenic variants of high-penetrance genes in patients with early-onset cancer, clinicians should be encouraged to consider performing multigene panel tests for these patients if their conventional *BRCA1/2* tests are negative.

This study has several limitations. The primary limitation is the small number of patients (*n* = 120), which provides only limited data for cancer susceptibility genes in Korean patients with breast cancer. A large-scale cohort study will be required to establish the accurate prevalence and spectrum of pathogenic variants in these patients. The majority of patients (87 of the 120, 72.5%) had VUS. A functional and population-based study will be necessary to clarify the clinical meaning of these VUS. Despite these limitations, to the best of our knowledge, this is the first prospective study to apply customized multigene panels to *BRCA1/2* mutation-negative Korean patients with a high risk for HBOC. A recent study conducted by Couch et al. assessed the commercial multigene panel test results of 65,057 patients with breast cancer; however, the frequency, phenotypic association, and cancer risks related to each variant were analyzed among Caucasian women only [11]. Regarding diversity of prevalence of the genetic variants, more prospective studies will be required among diverse ethnic populations.

## Conclusions

Wider application of multigene panel tests that include high-penetrance cancer susceptibility genes, so-called “beyond *BRCA1/2* genes”, will likely provide clinically relevant information for some patients with high risk for hereditary cancer [1, 13, 21]. However, these panels can produce abundant and conflicting results in clinical practice. To efficiently utilize these data, clinical databases should be established with respect to ethnic backgrounds, and genetic results should be carefully applied for high-risk patients.

## Additional files

Additional file 1: Figures S1 and S2. This file includes the methods detecting pathogenic variants and lage deletion in this study; depth of coverage and method for detection of large insertion-deletion of exon using next-generation sequencing, and confirmation of deleterious mutations using Sanger sequencing or MLPA in four patients. (PDF 1477 kb)

Additional file 2: Tables S1 and S2. This file includes two tables regarding baseline characteristics of study participants, possibly pathogenic variants and the classification according to ACMG guidelines mentied in the main manuscript. (DOCX 24 kb)

## Abbreviations

ACMG: American College of Medical Genetics and Genomics
AJCC: American Joint Committee on Cancer
BAM: Binary alignment map
BARD1: BRCA1-associated RING domain 1
BRIP1: BRCA1-interacting protein C-terminal helicase 1
CHEK2: Cell cycle checkpoint kinase 2
CI: Confidence interval
CNV: Copy number variants
ExAC: Exome Aggregation Consortium
F-CSGE: Fluorescent-conformation sensitive gel electrophoresis
HBOC: Hereditary breast and ovarian cancers
MLPA: Multiplex ligation-dependent probe amplification
NGS: Next generation sequencing
OR: Odds ratio
PM: Pathogenic criterion weighted as moderate
PP: Pathogenic criterion weighted as supporting
PVS: Pathogenic criterion weighted as very strong
SAM: Sequence alignment map
VUS: Variants of unknown significance
