# Supplementary material for: Variants of cancer susceptibility genes in Korean BRCA1/2 mutation-negative patients with high risk for hereditary breast cancer
Source: BMC Cancer. 2018 Jan 16;18:83. doi: 10.1186/s12885-017-3940-y (PMC5769462; doi:10.1186/s12885-017-3940-y)
Supplement: Supplementary file 1 — This file includes the methods detecting pathogenic variants and lage deletion in this study; depth of coverage and method for detection of large insertion-deletion of exon using next-generation sequencing, and confirmation of deleterious mutations using Sanger sequencing or MLPA in four patients. (PDF 1477 kb) [file 12885_2017_3940_MOESM1_ESM.pdf]

Fig S1. Depth of coverage and method for detection of large insertion-deletion of exon using next-generation sequencing

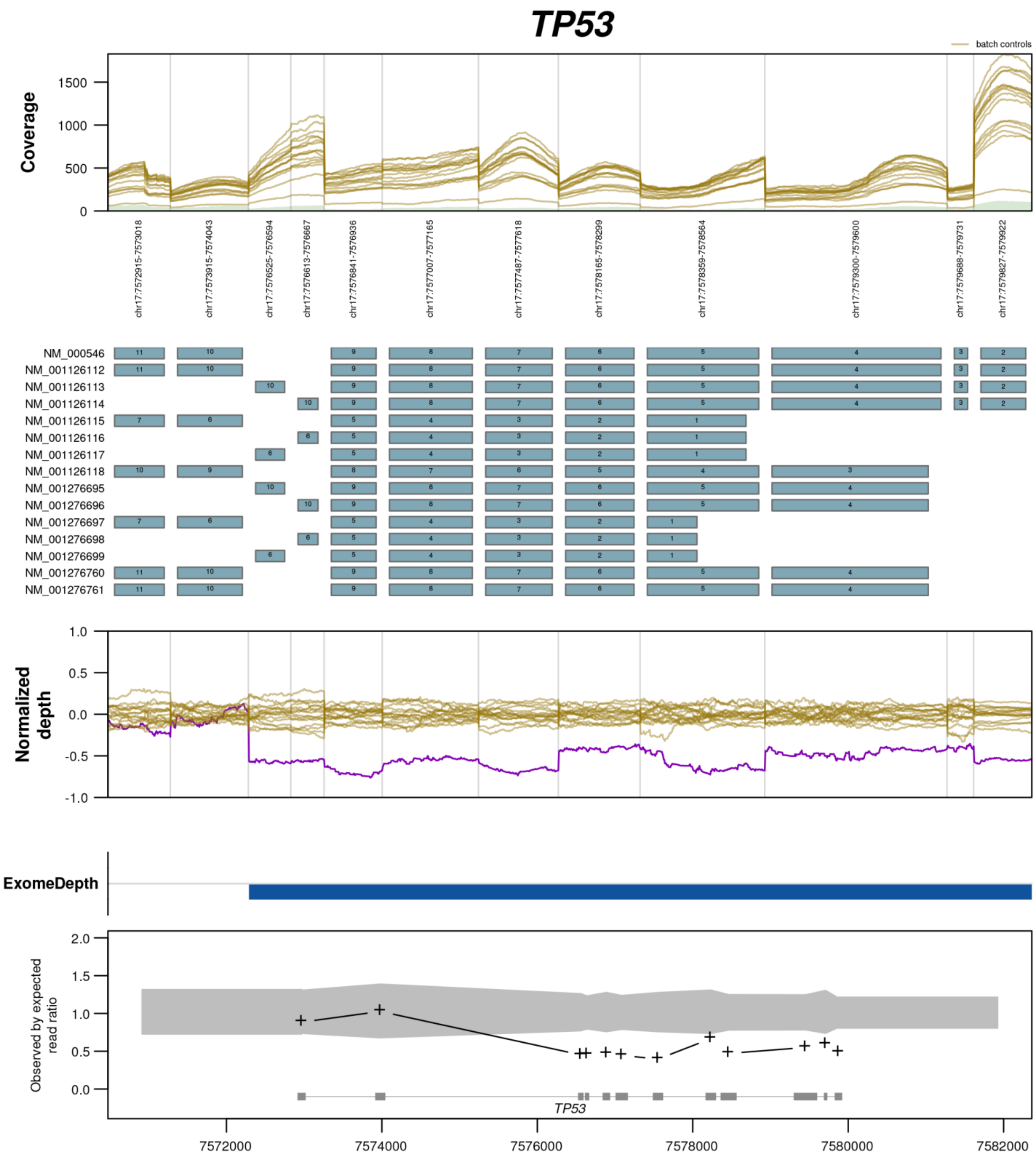

Fig S2. Confirmation of deleterious mutations using Sanger sequencing or MLPA; (A) case 2; (B) case 3; (C) case 7; (D) case 9

(A)

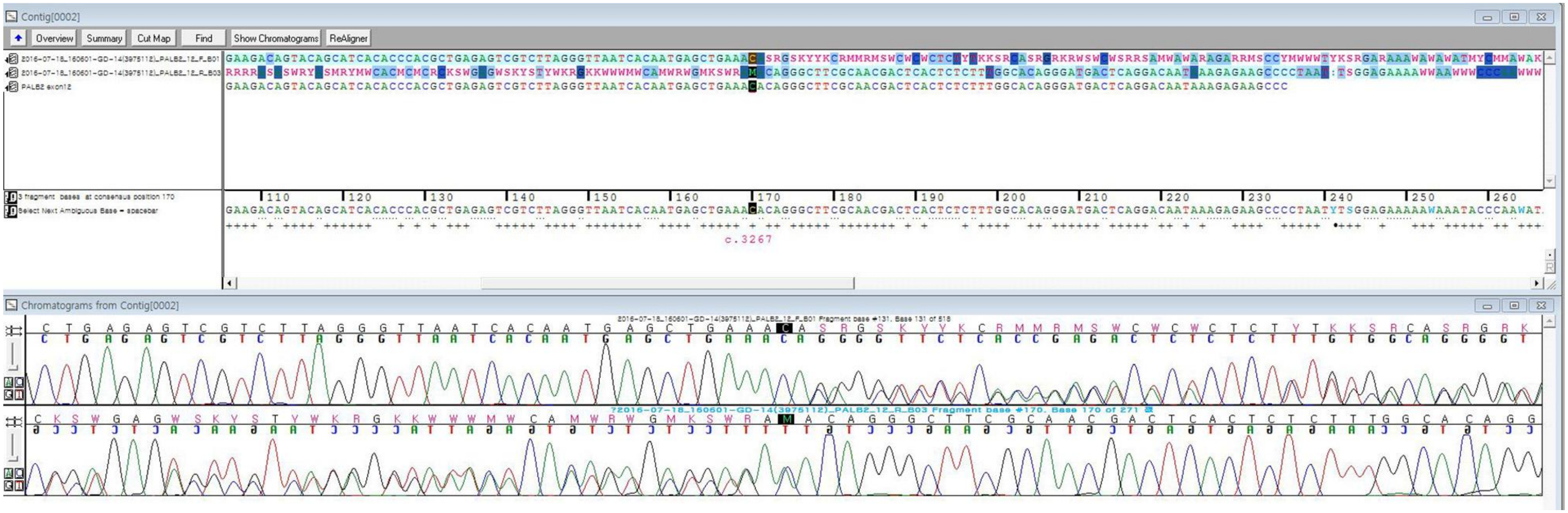

(B)

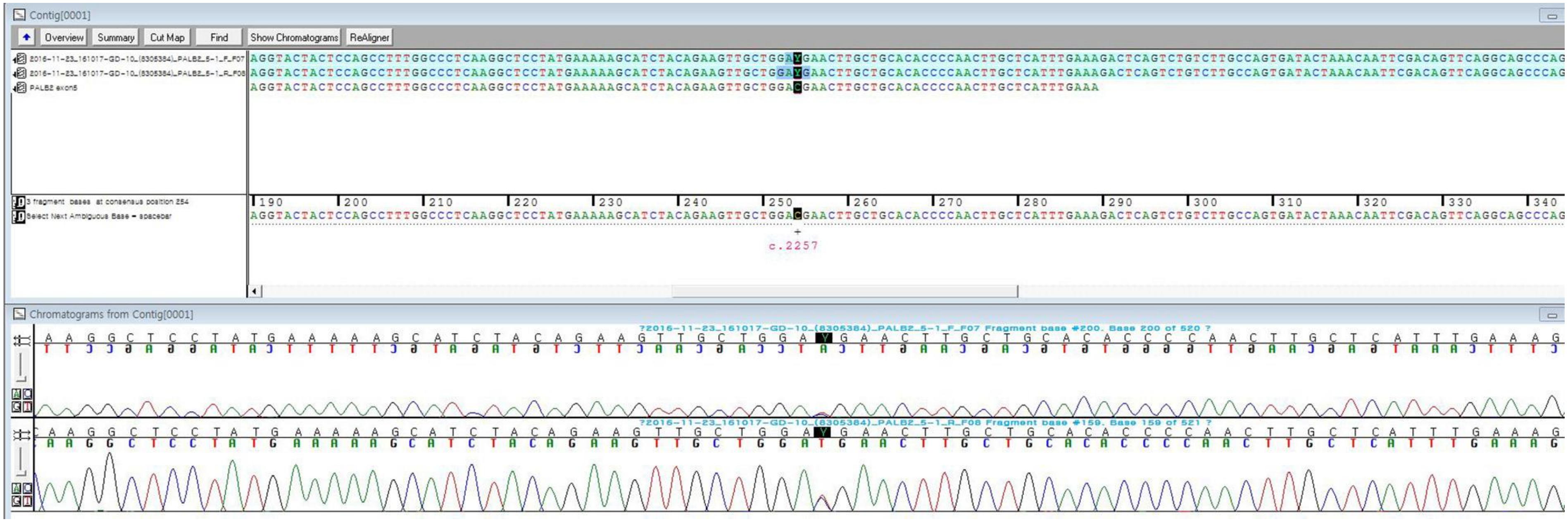

(C)

MLPA Analysis Report - SoftGenetics	
Software: GeneMarker V2.4.0	Analysis Type: MLPA
Project: Untitled	Compare Type: MLPA Ratio
Technician:	Normalization By: Population Normalization (Adjusted)
Report Time: 12/30/2016 - 17:13:43	Quantification By: Peak Height
Panel: P240_A3_BRIP1	Classification: Loss < 0.65 <= Equivalent <= 1.30 < Gain
Control: 2016-12-30_NP16(CGW,XY)_P240_H02.fsa	Report Value Type: Peak Ratio

2016-12-30_161031-GD-06_(8341731)_P240_F02.fsa

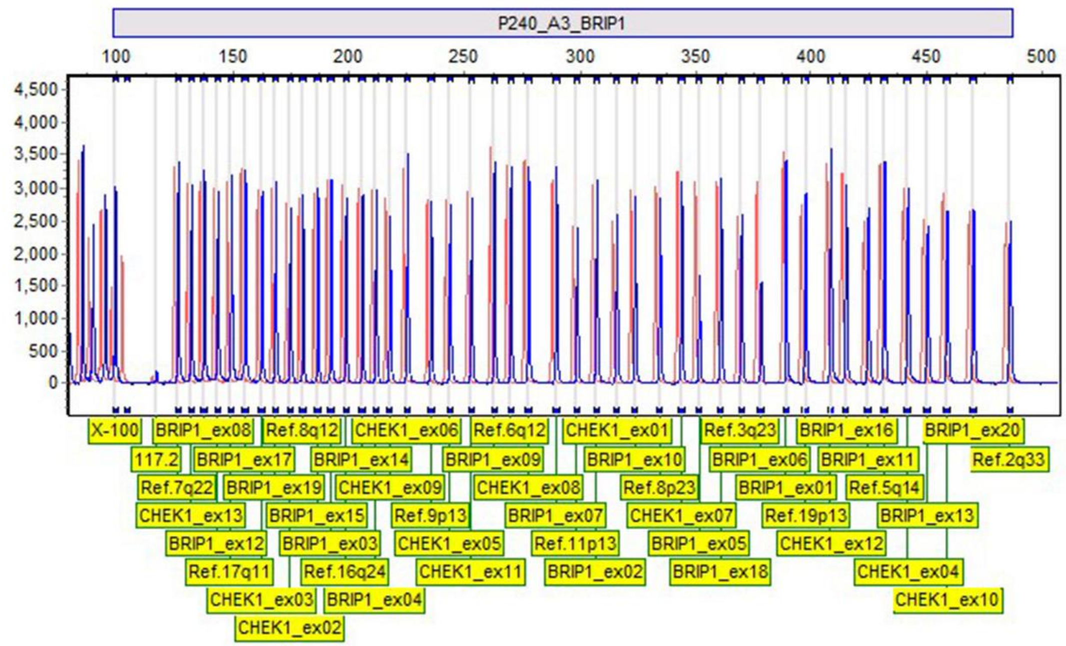

Sample Name: 161031-GD-06_(8341731)_P240
Machine: GA3130-1601-002
Run Time: 12/30/2016 - 13:51:37 -> 12/30/2016 - 14:43:27

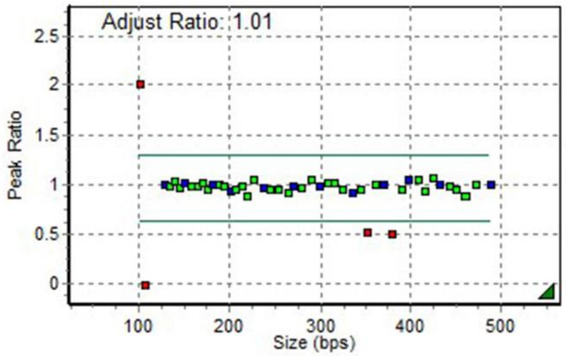

Conclusion		
	Date	Initial
Authorization		
Authorization		

	Probe Name	Bin Size	2016-12-30_161031-GD-06_(8341731)
1	X-100	100.0	2.032
2	Y-105	105.0	0.000
3	**Ref.7q22**	126.7	1.020
4	CHEK1_ex13	132.3	0.994
5	BRIP1_ex08	137.8	1.055
6	BRIP1_ex12	143.8	0.988
7	**Ref.17q11**	149.7	1.028
8	BRIP1_ex17	155.6	0.995
9	CHEK1_ex03	162.8	0.993
10	BRIP1_ex19	168.8	1.030
11	CHEK1_ex02	175.1	0.970
12	**Ref.8q12**	180.5	1.021
13	BRIP1_ex15	187.0	1.021
14	BRIP1_ex03	192.6	1.002
15	**Ref.16q24**	199.2	0.939
16	BRIP1_ex14	206.0	0.964
17	BRIP1_ex04	212.1	0.997
18	CHEK1_ex09	218.0	0.899
19	CHEK1_ex06	225.6	1.062
20	**Ref.9p13**	235.8	0.984
21	CHEK1_ex05	244.0	0.970
22	CHEK1_ex11	253.3	0.960
23	BRIP1_ex09	263.2	0.937
24	**Ref.6q12**	270.2	0.995
25	CHEK1_ex08	277.9	0.976
26	BRIP1_ex07	289.9	1.063
27	**Ref.11p13**	298.9	0.991
28	BRIP1_ex02	307.3	1.027
29	CHEK1_ex01	315.9	1.036
30	BRIP1_ex10	323.9	0.967
31	**Ref.8p23**	334.5	0.935
32	CHEK1_ex07	343.8	0.955
33	BRIP1_ex05	351.6	0.532
34	BRIP1_ex18	360.8	1.012
35	**Ref.3q23**	369.8	1.006
36	BRIP1_ex06	378.1	0.504
37	BRIP1_ex01	389.4	0.969
38	**Ref.19p13**	397.9	1.070
39	CHEK1_ex12	408.8	1.068
40	BRIP1_ex16	415.4	0.946
41	BRIP1_ex11	425.0	1.084
42	**Ref.5q14**	431.9	1.007
43	CHEK1_ex04	442.0	0.995
44	BRIP1_ex13	450.6	0.963
45	CHEK1_ex10	459.1	0.903
46	BRIP1_ex20	470.4	1.007
47	**Ref.2q33**	486.3	1.009

(D)

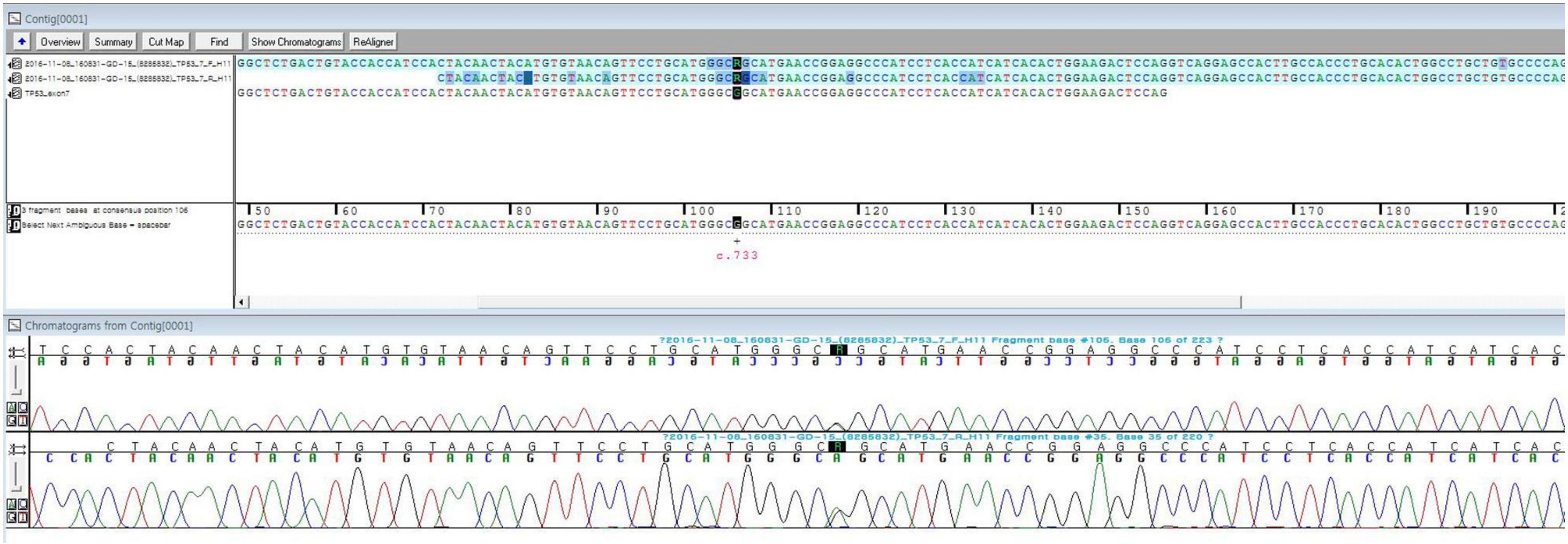
Contig[0001]
Overview
Summary
Cut Map
Find
Show Chromatograms
ReAligner
TP53_exon7
3 fragment bases at consensus position 106
Select Next Ambiguous Base = spacebar
c.733
Chromatograms from Contig[0001]
