# Supplementary material for: Variants of cancer susceptibility genes in Korean BRCA1/2 mutation-negative patients with high risk for hereditary breast cancer
Source: BMC Cancer. 2018 Jan 16;18:83. doi: 10.1186/s12885-017-3940-y (PMC5769462; doi:10.1186/s12885-017-3940-y)
Supplement: Supplementary file 2 — This file includes two tables regarding baseline characteristics of study participants, possibly pathogenic variants and the classification according to ACMG guidelines mentied in the main manuscript. (DOCX 24 kb) [file 12885_2017_3940_MOESM2_ESM.docx]

**Table S1.** Baseline characteristics of study participants

| **Characteristics** | | **Study participants (n=120)** | |
| --- | --- | --- | --- |
|  |  | number of participants | % |
| Age at first diagnosis of breast cancer (years) | | |  |
|  | median (range) | 42 (25–72) |  |
| Location | |  |  |
|  | Right | 53 | 44.2 |
|  | Left | 56 | 46.7 |
|  | Bilateral | 11 | 9.2 |
| Pathology | |  |  |
|  | IDC | 87 | 72.5 |
|  | DCIS | 16 | 12.5 |
|  | ILC | 6 | 5.0 |
|  | LCIS | 2 | 1.7 |
|  | mucinous | 7 | 5.8 |
|  | others | 3 | 2.5 |
| Hormone/ HER2 status | |  |  |
|  | ER+/ HER2- | 69 | 57.5 |
|  | ER+/ HER2+ | 6 | 5.0 |
|  | ER-/ HER2+ | 20 | 16.7 |
|  | TNBC | 21 | 18.3 |
|  | Unknown | 3 | 2.5 |
| Stage of disease | |  |  |
|  | 0 | 16 | 13.3 |
|  | I | 57 | 47.5 |
|  | II | 39 | 32.5 |
|  | III | 7 | 5.8 |
|  | IV | 1 | 0.8 |
| Other cancers concomitantly diagnosed with breast cancer | | |  |
|  | Gastric cancer | 3 | 2.5 |
|  | Thyroid cancer | 3 | 2.5 |
|  | Ovarian cancer | 3 | 2.5 |
|  | Ampulla of Vater cancer | 1 | 0.8 |
|  | Endometrial cancer* | 1 | 0.8 |
|  | Uterine cervix cancer | 1 | 0.8 |
| Family history of cancer | |  |  |
|  | 1st degree with breast/ ovarian cancer | 51 | 42.5 |
|  | 2nd degree with breast/ ovarian cancer | 14 | 11.7 |
|  | other cancer history | 43 | 35.8 |
|  | none | 12 | 10.0 |

Abbreviation: DCIS: ductal carcinoma in situ; ER: estrogen receptor; HER2: human epidermal growth factor receptor 2; IDC: invasive ductal carcinoma; ILC: invasive lobular carcinoma; LCIS: lobular carcinoma in situ; TNBC: triple negative breast cancer; *One patients with endometrial cancer was also diagnosed with ovarian cancer.

**Table S2.** Possibly pathogenic variants and the classification according to ACMG guidelines

| **Gene** | **Accession** | **DNA level change according to HGVS nomenclature^†^** | **Effect on amino acids^†^** | **dbSNP ID** | **Type** | **No. of patients (frequency) [total=120]** | **Global MAF by ExAC^‡^ [n=53,105]** | **Korean MAF by KRGBD* [n=622]** | **SIFT^§^** | **Polyphen-2^‖^** | **ClinVar^¶^** number of submissions (clinical significance) | **ACMG criteria^#^** | **ACMG  classification^#^** |
| --- | --- | --- | --- | --- | --- | --- | --- | --- | --- | --- | --- | --- | --- |
| TP53 | NM_000546.5 | c.733G>A | p.Gly245Ser | rs28934575 | Missense | 1 (0.0083) | 8.24x10^-6^ | <0.0016 | Probably damaging | Not tolerated | 7 (pathogenic/ likely pathogenic) | PM2, PM5, PP2, PP3, PP5 | Likely pathogenic |
| CHEK2 | NM_007194.3 | c.908+2delT | – | – | Splicing | 1 (0.0083) | – | – | N/A | N/A | N/A | PVS1 | Uncertain significance |
| CHEK2 | NM_007194.3 | c.1111C>T | p.His371Tyr | rs531398630 | Missense | 3 (0.025) | 0.0002 | 0.0038 | Probably damaging | Benign | 3 (conflicting interpretations;  likely pathogenic[1];  uncertain significance[2]) | PS4 | Uncertain significance |

Abbreviation: MAF, minor allele frequency;

**^†^**according to Human Genome Organisation (HUGO)-approved nomenclature (<http://www.hgvs.org/mutnomen/>)

**^‡^**from Exome Aggregation Consortium

*****from KRGDB (Korean Reference Genome DB; <http://152.99.75.168/KRGDB/>)

**^§^**using SIFT (Sorting Intolerant From Tolerant; <http://sift.jcvi.org/>);

**^‖^**using PolyPhen-2 (<http://genetics.bwh.harvard.edu/pph/>)

**^¶^**from National Center for Biotechnology Information (NCBI) ClinVar resources (<http://www.ncbi.nlm.nih.gov/clinvar/>)

**^#^**using American College of Medical Genetics and Genomics guideline (Richard et al., Genet in Med 2015)
